# Intermittent high-dose treatment with erlotinib enhances therapeutic efficacy in EGFR-mutant lung cancer

**DOI:** 10.18632/oncotarget.6276

**Published:** 2015-11-02

**Authors:** Jakob Schöttle, Sampurna Chatterjee, Caroline Volz, Maike Siobal, Alexandra Florin, Dennis Rokitta, Yvonne Hinze, Felix Dietlein, Dennis Plenker, Katharina König, Kerstin Albus, Johannes M. Heuckmann, Daniel Rauh, Thomas Franz, Bernd Neumaier, Uwe Fuhr, Lukas C. Heukamp, Roland T. Ullrich

**Affiliations:** ^1^ Department I of Internal Medicine, Center of Integrated Oncology Köln-Bonn, University of Cologne, Germany and Center of Molecular Medicine Cologne (ZMMK), University of Cologne, Cologne, Germany; ^2^ Max-Planck-Institute for Metabolism, with Klaus-Joachim-Zülch Laboratories of the Max Planck Society and the Medical Faculty of the University of Cologne, Cologne, Germany; ^3^ Department of Translational Genomics, University of Cologne, Medical Faculty, Weyertal, Cologne, Germany; ^4^ Department of Pathology, University Hospital Medical Center, University of Cologne, Cologne, Germany; ^5^ Department of Pharmacology, University Hospital Medical Center, University of Cologne, Cologne, Germany; ^6^ Max-Planck-Institute for Ageing, Cologne, Germany; ^7^ NEO New Oncology AG, Cologne, Germany; ^8^ Technical University Dortmund, Dortmund, Germany; ^9^ Institut für Radiochemie und Experimentelle Molekulare Bildgebung (IREMB), University Hospital Medical Center, University of Cologne, Cologne, Germany; ^10^ Department of Radiation Oncology, Cox-7, Harvard Medical School, Massachusetts General Hospital, Boston, MA, USA

**Keywords:** lung cancer, NSCLC, EGFR, erlotinib, high-dose scheduling, PET

## Abstract

Treatment with EGFR kinase inhibitors improves progression-free survival of patients with EGFR-mutant lung cancer. However, all patients with initial response will eventually acquire resistance and die from tumor recurrence. We found that intermittent high-dose treatment with erlotinib induced apoptosis more potently and improved tumor shrinkage significantly than the established low doses. In mice carrying EGFR-mutant xenografts intermittent high-dose treatment (200 mg/kg every other day) was tolerable and prolonged progression-free survival and reduced the frequency of acquired resistance. Intermittent EGFR-targeted high-dose schedules induce more profound as well as sustained target inhibition and may afford enhanced therapeutic efficacy.

## INTRODUCTION

EGFR kinase inhibitors have become routine treatment for patients with *EGFR*-mutant lung cancer [1-4]. However, resistance will ultimately emerge, thereby limiting the overall efficacy of such treatment. Resistance may emerge due to EGFR second site mutations, mainly the T790M-*gatekeeper* mutation, which prevents binding of the typical quinazoline-based compounds [5-8], due to genome alterations such as amplification of *MET* [9-15] that activate phosphatidyl-inositide-3 kinase (PI3K) signaling, or by processes changing cellular differentiation [16]. Both the T790M mutation and *MET* amplification may exist in a subclone present at the time of therapy; thus, a proportion of cases of resistance are likely to occur due to clonal selection of such resistant subclones under therapy [17, 18].

It was recently proposed that high-dose pulses of kinase inhibitors lead to enhanced target suppression and eradication of tumor cells more effectively by more potent induction of apoptosis [19]. As a consequence, intermittent high-dose schedules were shown to enhance efficacy in *ERBB2*-amplified breast cancer [20] as well as in *BRAF*-mutant melanomas [21]. In a cancer evolution modeling approach an intermittent scheduling of erlotinib could prevent the appearance of resistance despite the presence of EGFR^T790M^ positive subclones ab initio [22, 23].

Since the duration of target suppression is likely to affect the efficacy of a given compound in addition to the magnitude of target inhibition [24, 25] we sought to determine, whether enhanced trough levels or peak plasma levels might be more relevant to offer enhanced therapeutic efficacy.

## RESULTS

### High dose pulses of erlotinib potently inhibit tumorcell growth of EGFR-mutant NSCLC cell lines *in vivo* and *in vitro*

**Figure 1:**
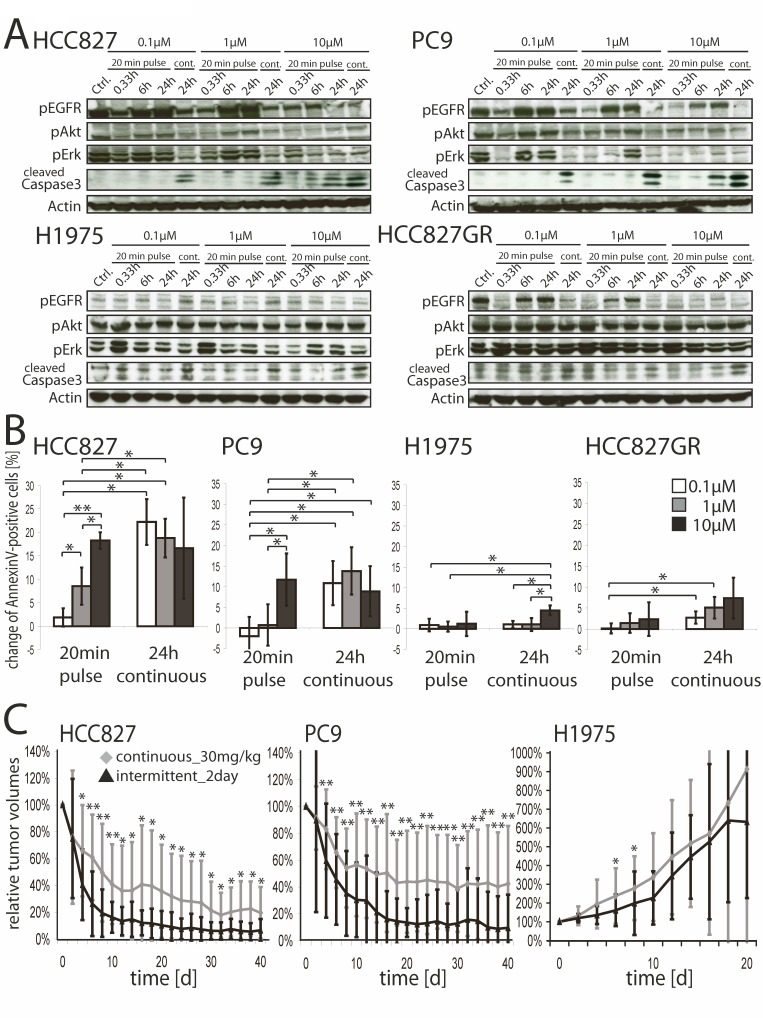
**A.** Western Blot analyses of HCC827, PC9, H1975 and HCC827GR cells treated with 0.1μM, 1μM or 10μM of erlotinib or DMSO for 20 minutes or continuously till preparation of lysates. Whole-cell lysates were analyzed for expression levels of the indicated proteins by western blotting. **B.** AnnexinV flow cytometry of HCC827, PC9, H1975 and HCC827GR treated with 0.1μM, 1μM, 10μMerlotinib or DMSO for 20 minutes or continuously. FACS-analysis was done 24 hours after initial exposure to erlotinib and read-out was normalized to DMSO-control. Change of Annexin V/PI-double positive cells ±SD are shown. *p < 0.05, **p < 0.001. **C.** shows relative tumor volumes of xenografts ±SD (HCC827, PC9 and H1975). Xenograft harboring mice were treated with 30mg/kg erlotinib daily or 200mg/kg erlotinib every 2^nd^ day p.o. and tumor volumes were measured every 2^nd^ day.*p < 0.05, **p < 0.001.

In line with a recent report [19] 20 minutes of treatment with 10μM of erlotinib potently suppressed tumor cell growth of the erlotinib sensitive cell lines HCC827 and PC9 (both carry EGFR exon 19 deletion mutations) as effectively as a continuous 72 hours exposure with 0.1μM. As expected, any such treatment had minor effect on the resistant cell lines H1975 (EGFR L858R and T790M) and HCC827GR (EGFR exon 19 deletion, *MET*-amplified) (Figure S1), thus confirming that efficacy was due to on-target activity of the compound. A pulse of 10μM of erlotinib reduced pAkt and pErk1/2 levels in the sensitive cell lines, despite re-establishment of pEGFR signaling (Figure 1A, upper panels). Continuous exposure to erlotinib inhibited pEGFR, pAkt and pErk1/2 levels at a concentration of 0.1μM. Again, downstream signaling was only slightly reduced in the two resistant control cell lines (Figure 1A, lower panels). Phosphorylated levels of MET were reduced in HCC827, PC9 and H1975 cells by high concentrations of erlotinib, but not in the *MET-*amplified cell line HCC827GR (Figure S2). Induction of apoptosis (Figure 1A, 1B) was also similar in the sensitive cells treated with a high-dose pulse of 10μM for 20 minutes and those treated with 0.1μM, but not in the resistant ones.

Given the high efficacy of high-dose treatment of erlotinib in *EGFR*-mutant tumor cells *in vitro*, we tested whether pulsatile high doses of erlotinib enhanced tumor control *in vivo*. Tumor shrinkage occurred more rapidly and was of greater magnitude in tumor xenografts of PC9 and HCC827 cells treated with intermittent high doses of erlotinib (200mg/kg every other day, “intermittent_2day”) than in those treated daily with 30mg/kg (Figure 1C). Treatment with either 15mg/kg daily or 200mg/kg every fourth day (“intermittent_4day”) were less effective, although this effect was more pronounced in PC9 xenografts (Figure S3). Again, H1975 was unresponsive to therapy using any of these schedules (Figure 1C and Figure S3, right panels).

### Toxicity as well as pharmacokinetic aspects of high-dose pulse treatment

**Figure 2:**
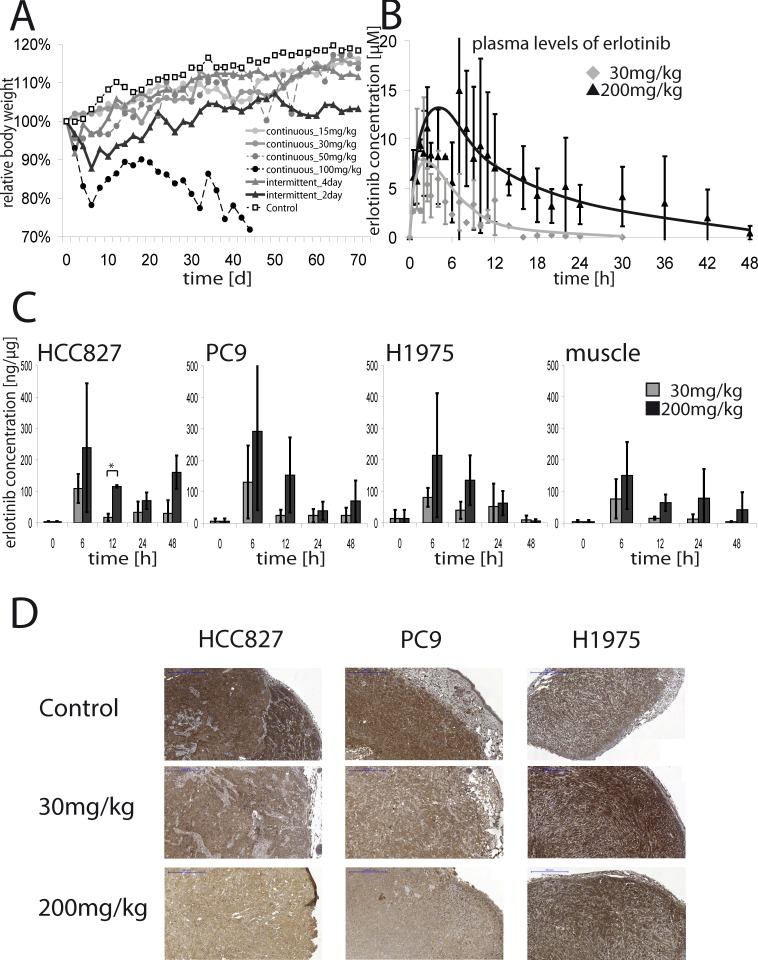
**A.** Relative body weight of mice treated witherlotinib15mg/kg daily, 30mg/kg daily, 50mg/kg daily, 100mg/kg daily, 200mg/kg every 4^th^ day, 200mg/kg every 2^nd^ day or vehicle detergent alone. Shown is the mean weight of mice, set relative to the weight at the beginning of the therapy. In **B**. mean plasma concentrations of erlotinib ±SD in mice are shown. Non-tumor harboring mice were treated orally with a single dose of either 30mg/kg or 200mg/kg and blood samples were taken from the tail-vein. Plasma-concentrations were determined by liquid chromatography tandem mass spectrometry. **C.** Mean erlotinib concentrations ±SD in tumor lysates (HCC827, PC9, H1975) or lysates of muscle tissue of mice treated with a single dose of either 30mg/kg or 200mg/kg erlotinib are shown. Lysates were prepared from untreated mice or 6, 12, 24 or 48 hours after administration of erlotinib. Erlotinib concentrations in the supernatant were assessed by mass spectrometry and set relative to the protein-amount of the lysate.*p < 0.05. **D.** Representative IHC-stainings for pEGFR of tumors (HCC827, PC9, H1975) of mice either untreated or treated with a single dose of 30mg/kg or 200mg/kg erlotinib. Tumors were resected 12 hours after treatment. 5x magnification, blue scale bar indicates 500μm.

Alignment of murine and human EGFR revealed only one different amino acid in the kinase domain (Y > F at 771), which is unlikely to affect binding of erlotinib (Figure S4A). Thus, erlotinib is expected to inhibit EGFR signaling in all murine tissues, in which EGFR is expressed. We found that mice treated with 100mg/kg erlotinib daily lost about 20% of weight. The intermittent_2day schedule led to an initial weight loss of 10%, too, but mice typically recovered within 20 days. Mice treated with 50mg/kg daily and the intermittent_4day schedule initially showed a slight reduction of weight of about 5%, but recovered within 10 days. Mice treated with 15mg/kg or 30mg/kg daily and the control group did not exhibit significant weight loss (Figure 2A). Both the 50mg/kg and 100mg/kg daily schedules were highly toxic: severe diarrhea occurred in 88% and 73% of mice, respectively. By contrast, diarrhea occurred in 49% of the mice in the intermittend_2day and 20% of the intermittend_4day groups (Figure S4B). In the 100mg/kg daily group significantly more mice died during therapy than in the other groups (Figure S4C). Rash occurred in 38% of mice in the 100mg/kg daily cohort, whereas rash was rare in the mice treated with the other schedules (Figure S4D). There was no case of diarrhea or rash in the control group. Thus, intermittent high-dose treatment of EGFR-mutant tumors with erlotinib enhanced tumor control with limited toxicity.

Estimates for erlotinib peak plasma concentrations after a single dose of 30mg/kg or 200mg/kg of erlotinib were 6.5μmol/l and 11.7μmol/l, respectively. The area under the curve (AUC) showed linear increase with the dose (3.84μmol*h *vs*. 24.45μmol*h (*p* < 0.001)). Thus, erlotinib clearance was independent on the dose (2.72l/h *vs*. 3.05l/h (*p* = 0.45)); however, the apparent absorption rate constant was much higher for the low dose (0.36/h *vs*. 0.08/h (*p* < 0.001)), suggesting some saturation of absorbtion of erlotinib in the intestine (Figure 2B). The concentrations of erlotinib in tumor lysates of HCC827, PC9 and H1975 xenografts peaked after 6 hours. In mice treated with 30mg/kg of erlotinib the peak concentrations reached about 100ng of erlotinib/μg of protein and declined after 12 hours. Treatment with 200mg/kg led to peak tumor tissue concentrations of 200-250ng/μg. In muscle tissue concentrations were lower, suggesting enrichment in tumor tissue (Figure 2C). The peak tumor concentrations of the active metabolite OSI-420 were about 10ng/μg for 30mg/kg erlotinib and declined completely within 24 hours, and reached 30 to 50ng/μg in tumor tissue and about 20ng/μg in muscle tissue for the 200mg/kg dose (Figure S5). We finally assessed the pharmacodynamic effects of high-dose treatment in tumors explanted from treated mice by pEGFR-immunohistochemistry. In the sensitive HCC827- and PC9-xenografts both 30mg/kg and 200mg/kg of erlotinib reduced pEGFR compared to untreated controls and the resistant H1975-xenografts. However single dosing of 200mg/kg of erlotinib reduced pEGFR much stronger than 30mg/kg (Figure 2D).

### *In vivo* pharmacodynamic assessments by 18F-FLT-PET

**Figure 3:**
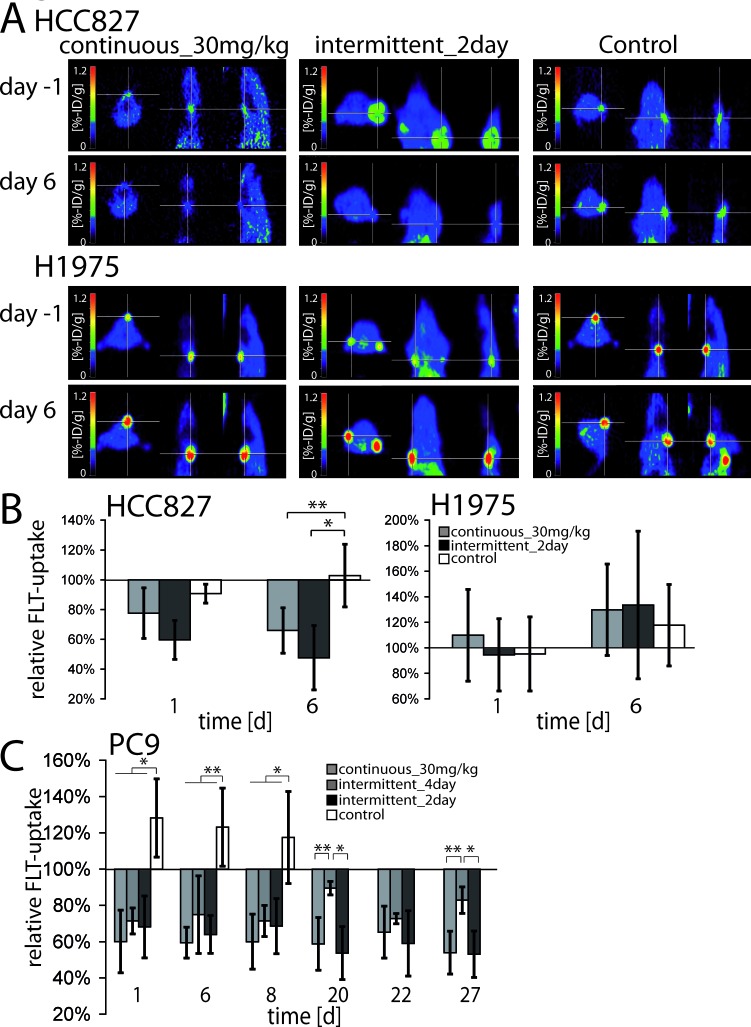
**In A.** representative ^18^F-FLT-images of mice harboring HCC827 or H1975 xenografts treated with 30mg/kg erlotinib daily, 200mg/kg erlotinib every 2^nd^ day or vehicle are shown. ^18^F-FLT-PET measurements were performed the day before start of therapy and at day 6 of therapy. The cross hairs indicate tumor positions. **B.** Change in relative ^18^F-FLT-uptake of HCC827- and H1975-xenografts. Mice were treated with either 30mg/kg erlotinib daily or 200mg/kg erlotinib every 2^nd^ day or vehicle. ^18^F-FLT-PET-imaging was performed the day before stat of therapy (day -1), day 1 and 6 after start of therapy. All values were set relative to day -1. Error bars indicate ±SD, *p < 0.05, **p < 0.001. **C.** Change in relative ^18F^FLT-uptake of PC9 xenografts. Mice were treated with either 30mg/kg erlotinib daily,200mg/kg erlotinib every 4^th^ day, 200mg/kg erlotinib every 2^nd^ day (up to day 27) or vehicle (up to day 8). ^18^F-FLT-PET- imaging was performed at day -1, day 1, 6, 8, 20, 22 and27 after start of therapy. Treatment days of the intermittent_4day schedule were: day 0, 4, 8, 12, 16, 20, 24, 28. All values were set relative to day -1. Error bars indicate ±SD.*p < 0.05, **p < 0.001.

We have recently shown in mice and in patients that erlotinib induces early cell cycle arrest in EGFR-mutant tumors that precedes induction of apoptosis and that can be monitored *in vivo* using ^18^F-FLT-PET [26, 27]. We therefore determined, whether the dynamics of induction of cell cycle arrest and tumor shrinkage might also be similar in the 30mg/kg daily and the intermittend_2day schedules. While in H1975 xenografts uptake of ^18^F-FLT was not reduced by erlotinib treatment (Figure 3A, lower panel, Figure 3B, right panel, Figure S6 and S7), the decrease in relative FLT-uptake was similar in both the continuous 30mg/kg and the intermittent_2day schedules in HCC827 and PC9 xenografts (Figure 3A, upper panel, Figure 3B left panel, Figure 3C and Figure S6 and S7). In PC9 tumors the intermittent_4day showed a similar decline in FLT-uptake at days 1, 6 and 8; however ^18^F-FLT-uptake increased again at days 20 and 27, but not in the continuous_30mg/kg and intermittent_2day group (*p* < 0.05) (Figure 3C). This observation corroborates the notion that both high trough and peak levels of erlotinib are relevant for cell cycle arrest [28, 29] and tumor shrinkage.

### Intermittent high-dose erlotinib treatment improves progression-free survival of mice bearing EGFR-mutant xenografts

**Figure 4:**
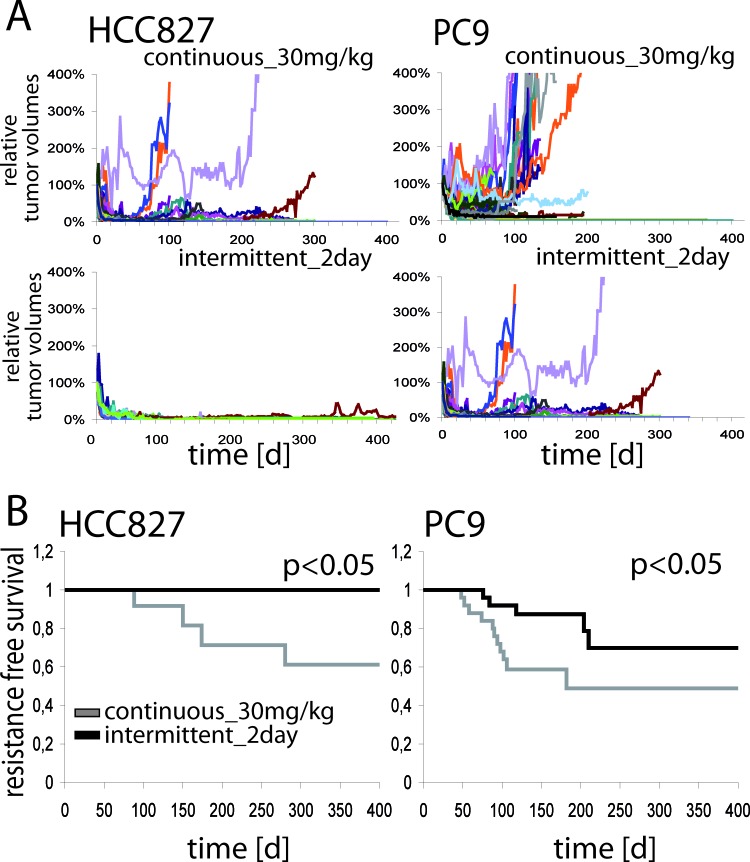
**A.** Tumor volumes of HCC827- (left panel) and PC9- (right panel) xenograft harboring mice treated long term with erlotinib either 30mg/kg daily (upper row) or 200mg/kg every 2^nd^ day (lower row). Each colored line represents the relative volume of one single tumor. **B.** Kaplan Meier curves of resistance free survival of mice harboring HCC827- (left) and PC9- (right) xenografts treated long term with erlotinib. Shown are the treatment schedules with 30mg/kg erlotinib daily or 200mg/kg erlotinib every 2^nd^ day. p indicates statistical significance by log-rank-test.

High doses of erlotinib improved tumor shrinkage by enhanced target signaling suppression. Efficacy of EGFR inhibitors is ultimately limited by the emergence of resistance. We therefore tested the hypothesis that intermittent high dose treatment of EGFR-mutant tumors might also enhance the duration of response. Mice engrafted with HCC827 or PC9 cells were therefore treated with the most effective, but still tolerable schedules for up to 400 days: 30mg/kg erlotinib daily or 200mg/kg erlotinib every other day. Median follow-up time for HCC827 was 280 (continuous_30mg/kg) and 400 (intermittent_2day) days and for PC9 102 and 160 days, respectively (Figure S9). All tumor volumes of these mice are individually shown in Figure 4A. After initial response several tumors restarted to grow. However, in the case of HCC827 xenografts treated with 200mg/kg every second day no resistance emerged, whereas 40% of the tumors treated with 30mg/kg daily became resistant (*p* < 0.05). PC9-xenografts showed acquired resistance in both therapy groups; however, recurrence occurred later (Figure 4A, right) and progression-free survival was longer in the group receiving intermittent high-dose treatment (*p* < 0.05) (Figure 4B).

Sequencing of tumors explanted at the time of resistance revealed the T790M resistance mutation in PC9 xenografts (Figure S10). These results show that more pronounced target suppression not only leads to higher magnitude, but also greater duration of response.

## DISCUSSION

Here we present a strategy for improving therapeutic efficacy in a xenograft model of *EGFR*-mutant lung cancer by intermittent high-dose scheduling of erlotinib. This schedule was tolerable, but enhanced both the magnitude and the duration of response. Despite the high dose, efficacy was due to on-target effects of erlotinib as the treatment effects were not observed in cells bearing the T790M resistance mutation.

We could show that plasma peak levels of erlotinib after a dose of 200mg/kg were only two fold higher than after 30mg/kg indicating saturation of absorption. By contrast, the AUC was about 6-fold higher suggesting that the duration of target suppression was increased several-fold. As a consequence we found for pulsatile high-dose treatment of erlotinib stronger and more durable target suppression by pEGFR-staining, and could show sufficient cell-cycle-arrest in-vivo by ^18^F-FLT-PET.

Consequently, intermittent high-dose treatment improved the progression-free survival in two EGFR-mutant xenografts in a long-term treatment study. While emergence of resistance was completely abolished in HCC827 xenografts, resistance occurred less frequently and at a later time in PC9 xenografts. The latter are known to become resistant to erlotinib over time due to emergence of the T790M resistance mutation; thus, the efficacy of reversible quinazoline EGFR inhibitors will always be limited in these tumors. In line with these findings we could detect the T790M mutation in resistant PC9 tumors, but not in HCC827 tumors. However, the observation that PFS could still be improved in PC9 tumors may arguably be due to the possibility that high doses of erlotinib are still capable of inhibiting T790M-mutant EGFR to some extent (note that the biochemical IC50 of erlotinib for EGFR^T790M^ is still in the nanomolar range).

In a first study in humans treated with an intermittent high-dose schedule of erlotinib, patient suffered only moderate side-effects [30]. The limited efficacy in this study is likely due to the lack of patient selection based on *EGFR* mutations. Thus, patients with EGFR-mutant lung cancer may benefit from high-dose treatment with EGFR inhibitors at reasonable toxicity. In line with this notion, Riely and colleagues showed in a recent phase II study, that OS of patients can be prolonged by a high-dose pulse of erlotinib before chemotherapy, without increasing toxic side effects [31]. Patients on this trial were not selected based on *EGFR* mutations either. Thus, patients with *EGFR*-mutant lung cancer may derive further benefit from such intermittent high-dose schedule. However, in these studies no pharmacokinetic analyses in patients treated with high-dose erlotinib were performed. Further studies are required to determine peak and AUC levels during high dose erlotinib treatment and to evaluate whether these high dose erlotinib levels in mice are tolerable in humans. Finally, these high doses of erlotinib may penetrate the blood-brain barrier and induce tumor shrinkage in *EGFR*-mutant brain metastases, which are usually not tractable by standard dosing of erlotinib [32].

In summary, we provide evidence that intermittent high-dose treatment of *EGFR*-mutant tumors with erlotinib enables enhanced tumor shrinkage and prolonged PFS, while limiting toxicity in a mouse xenograft study. This effect is associated with high trough and peak levels, which induced more durable shutdown of *EGFR*-associated oncogenic signaling. While treatment is increasingly tailored to each patient based on tumor genotyping, the established 150mg once daily dose of erlotinib may simply not be the ideal schedule for optimal target shutdown and tumor control. Thus, protocols testing such intermittent high dose pulses of erlotinib specifically in patients with *EGFR*-mutant lung cancer warrant clinical exploration.

## MATERIALS AND METHODS

### Cell-culture and reagents

The human NSCLC celllines PC9, HCC827 and H1975 were obtained from the American Type Culture Collection (ATCC), HCC827GR were kindly provided by the laboratory of Jeff Engelman. Cells were cultured in RPMI-1640 medium with 10% FCS and 1% Penicillin+Streptomycin.

Erlotinib was purchased from LC Labs, USA. For in-vitro studies erlotinib was prepared in stock solution of 10mM in DMSO (Sigma Aldrich, Germany) and stored at −20°C. For in-vivo studies erlotinib was dissolved in 6% Captisol^®^ (CyDex Inc., USA) in concentrations of 10mg/ml (continuous schedules) or 30mg/ml (intermittent schedules) and stored in a rotating device at 4°C.

### Immunoblotting

Immunoblotting was performed using the following antibodies: pEGFR (Y1068), EGFR, pAkt (S473), AKT, pErk 44/42 (Thr202/Tyr204), ERK 44/42, Caspase-3 (Cell Signaling Technologies, USA), Δ-actin (clone C4) (MP Biomedicals LLC, USA), anti-rabbit-HRP- and anti-mouse-HRP-antibody (Millipore, Germany).

### Annexin V-flow-cytometry

Flow cytometryofHCC827, PC9, H1975 and HCC827GR cells treated with erlotinib (0.1μM, 1μM or 10μM) or DMSO for either 20 minutes or 24 hours, was performed using the AnnexinV-FITC Apoptosis Detection Kit I (BD Pharmingen, Germany) according to the manufactures protocol and measured by Gallios Flow Cytometer (Beckmann Coulter), detecting at least 100,000 events per probe. Data was evaluated by setting appropriate gates in Kaluza analysis software (Beckman Coulter) and apoptosis was calculated as the difference between sample and DMSO control.

### *In vivo* experiments

All animal procedures were approved by the local animal protection committee and the local authorities.

All experiments were performed in 10 to 15 week old male athymic NMRI-nude-mice (Janvier, Europe) as described recently [26, 33]. For creation of xenografts tumor cells (HCC827, PC9 and H1975) were implanted subcutaneously. Therapy was started when tumors reached a size of approx. 100mm³. Mice were treated by oral gavage of erlotinib in these schedules: 15mg/kg daily, 30mg/kg daily, 200mg/kg every 2^nd^ day or 200mg/kg every 4^th^ day or 100μl vehicle detergent daily.

Tumor size was monitored every 2^nd^ day by calimetric measurement. Tumor volumes were calculated by the modified ellipsoid formula [V = 0.5 × (long diameter) × (short diameter)²;]. All absolute tumor volumes were set relative to day 0.

### Toxicity of erlotinib in mice

Human and murine EGFR were aligned using the ClustalW-alignment (http://www.ebi.ac.uk/Tools/msa/clustalw2) and performed an analysis of erlotinib binding to the ATP-binding pocket of the EGFR. To assess the tolerable therapy schedules of erlotinib we treated non-tumor harboring mice with these schedules of erlotinib: 15mg/kg, 30mg/kg, 50mg/kg or 100mg/kg daily, or with 200mg/kg every 2^nd^ or 4^th^ day, as well as with vehicle detergent alone daily. Weight was measured every 2^nd^ day and toxic side effects (diarrhea, rash and death) in all treated mice were monitored.

### Pharmacokinetics of erlotinib

For pharmacokinetic analyses mice were treated with a single dose of either 30mg/kg or 200mg/kg erlotinib. Blood samples were taken from the tail-vein and erlotinib plasma concentrations were quantified using liquid chromatography tandem mass spectrometry with a lower limit of quantification of 18ng/ml.

### Mass spectrometry of erlotinib from tumor lysates

For determination of erlotinib concentration in the tumor, HCC827, PC9 and H1975 xenografts were used. Mice were treated with a single dose of either 30mg/kg or 200mg/kg erlotinib and sacrificed 6, 12, 24 or 48 hours after treatment or without treatment. Then tumors were resected and lysed for determination of erlotinib and OSI-420 concentrations within the tumor by mass spectrometry. Erlotinib and OSI-420 concentration in the lysates were set relative to the protein concentration of the lysate.

### Immunohistochemistry

For pEGFR-immunohistochemistry tumors were resected 12 hours after treatment with either 30mg/kg or 200mg/kg erlotinib or without treatment. Tumors were fixed in 4% formaldehyde for 24 hours and transferred to PBS. Tissues were embedded in paraffin, were cut and stained with pEGFR Tyr 1068 primary antibodies (1:100 over night, pretreatment pH6 20min), Corresponding secondary antibody detection kits for reduced background on murine tissue were used (Histofine Simple Stain Mouse MAX PO and Histofinemousestain kit, medac) and stained on an automated stainer (LabVisionAutostainer 480S, Thermo Scientific).

### ^18^F-FLT-PET imaging

Synthesis of ^18^F-fluoro-L-thymidine (^18^F-FLT) and PET measurement protocols were performed as described elsewhere [26, 34, 35].

Mice harboring HCC827, PC9 and H1975 xenografts were measured one day before start of therapy (day -1), at the second day of therapy (d = 1) and at day 6 and 8 using a R4 microPET scanner (Concord Microsystems, Inc, Knoxville, TN). Quantitative analysis was done using the in-house software VINCI using a region of interest (ROI) analysis. All data were decay corrected.

### Statistical analysis

For statistical analyses we used Sigma Plot 11.0 (Systat Software, USA). We used student's *t*-test (unpaired, 2-sided), χ^2^-test and log-rank-test. *p* < 0.05 was considered statistical significant.

For further details we refer to Supplementary Methods.

## SUPPLEMENTARY MATERIAL FIGURES AND METHODS


