# Supplementary material for: Intermittent high-dose treatment with erlotinib enhances therapeutic efficacy in EGFR-mutant lung cancer
Source: Oncotarget. 2015 Nov 2;6(36):38458–68. doi: 10.18632/oncotarget.6276 (PMC4770714; doi:10.18632/oncotarget.6276)
Supplement: Supplementary file 1 [file oncotarget-06-38458-s001.pdf]

Intermittent high-dose treatment with erlotinib enhances therapeutic efficacy in EGFR-mutant lung cancer

Supplementary Material

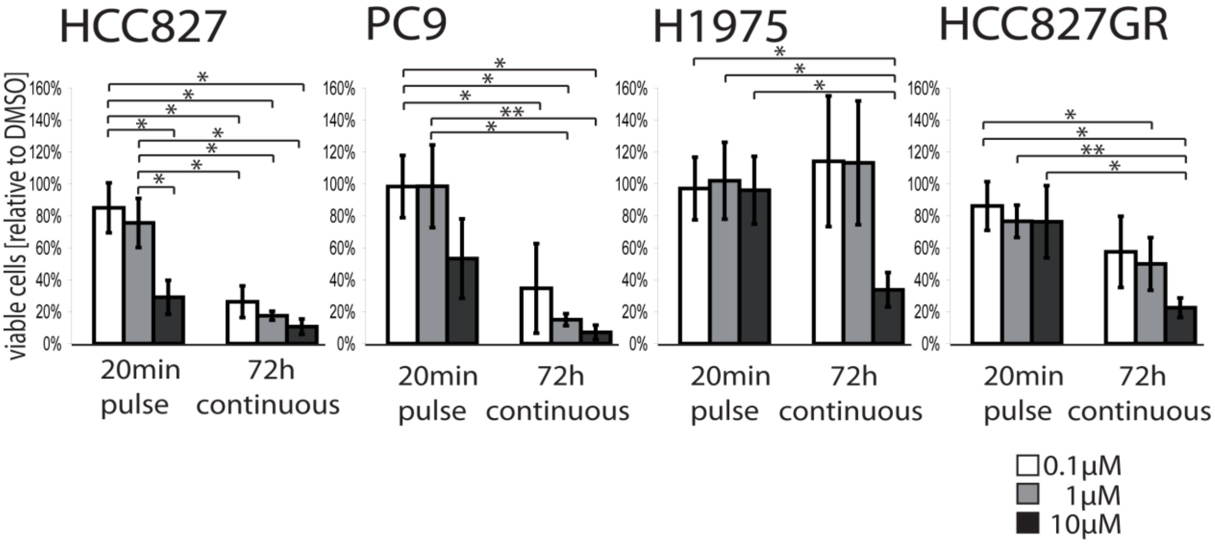

Figure S 1

Cell viability of HCC827, PC9, H1975 and HCC827GR cells assessed by MTT-Assay. Plated cells were treated with 0.1µM, 1µM, 10µM erlotinib or DMSO in RPMI-1640 medium for 20 minutes or 72 hours. After 20 minutes of treatment medium was removed, cells were washed 3 times and fresh medium containing either DMSO or erlotinib was used. After 72 hours total treatment time MTT-Assay was performed. Mean data normalized to DMSO ±SD is shown, *$p < 0.05$, **$p < 0.001$.

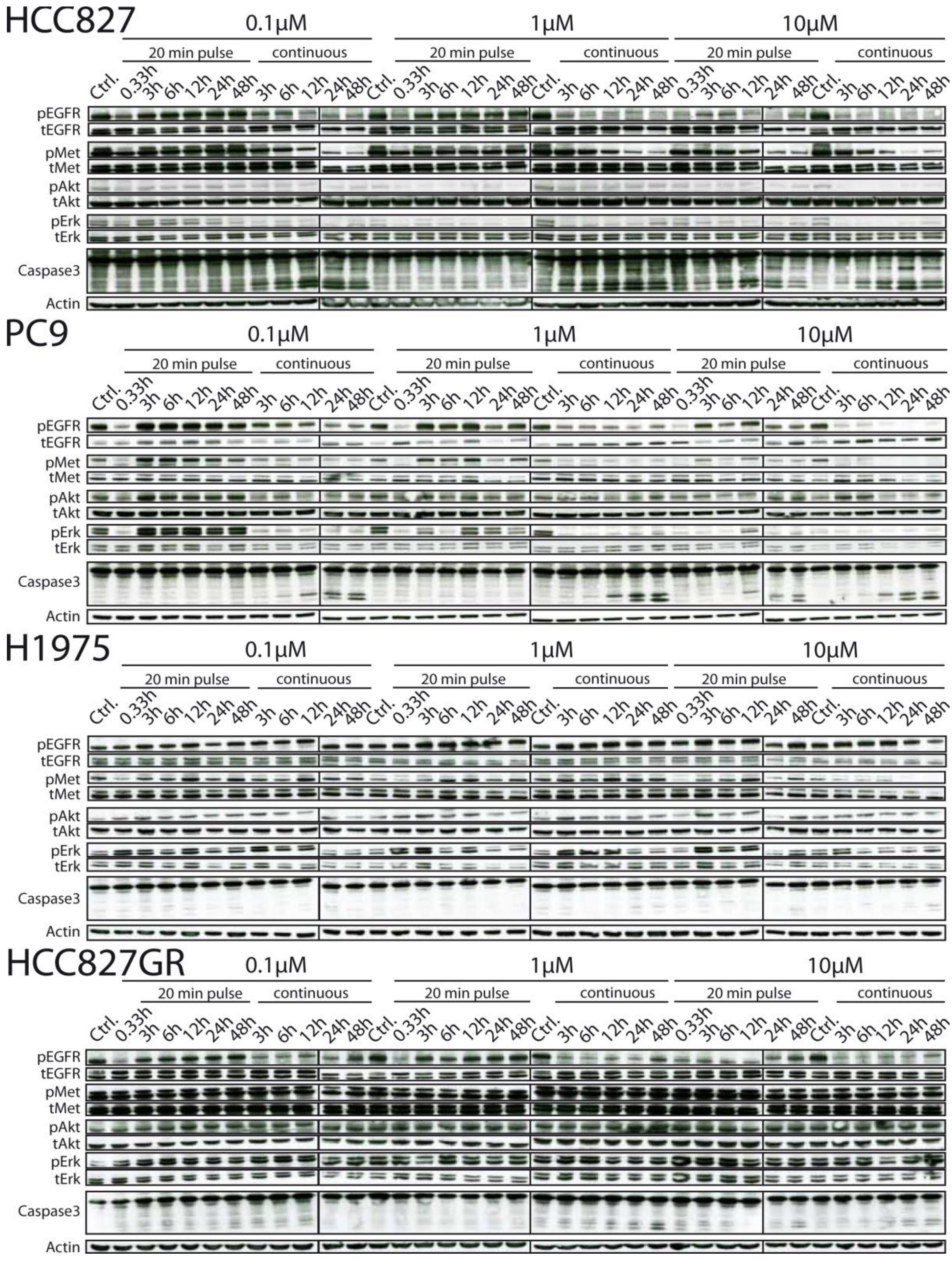

Figure S 2

Western Blot analyses of HCC827, PC9, H1975 and HCC827GR-cells treated with 0.1µM, 1µM, 10µM erlotinib or DMSO with a 20 minutes pulse or up to 48 hours continuously. Cell lysates were prepared after 20 minutes, 3, 6, 12, 24 and 48 hours, Whole-cell lysates were analyzed for expression levels of the indicated proteins by western blotting: pEGFR, EGFR, pMet, Met, pAkt, Akt, pErk1/2, Erk1/2, Capase3, Actin.

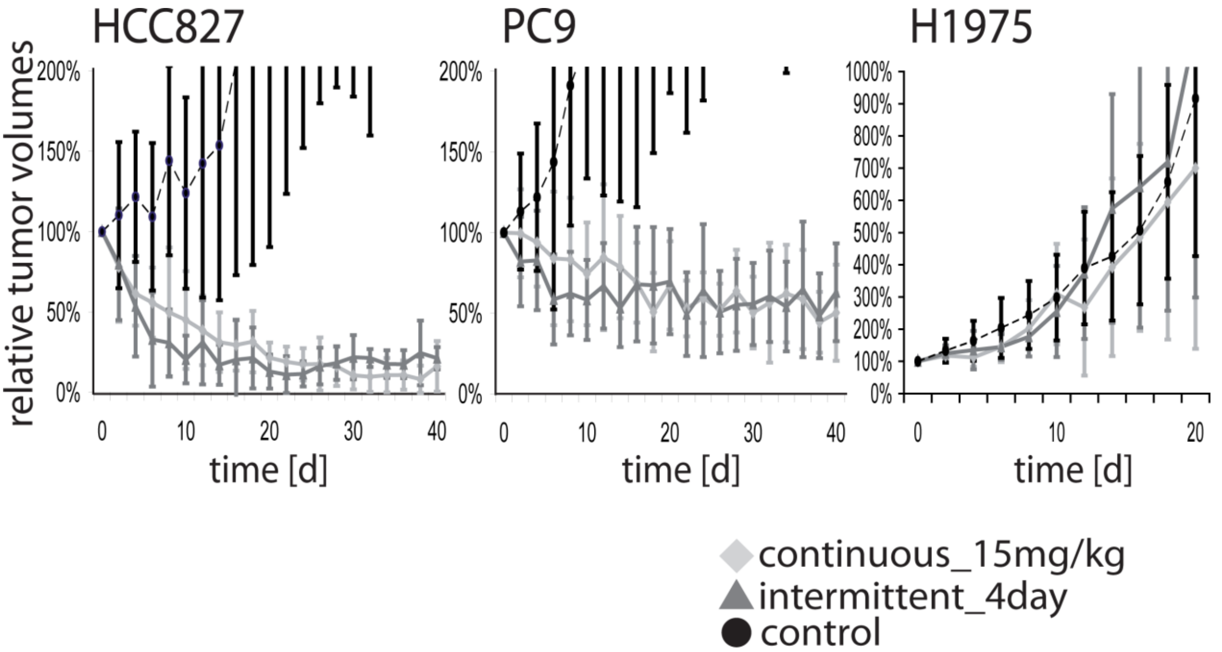

Figure S 3

Relative tumor volumes ±SD of xenografts treated with erlotinib 15mg/kg daily, 200mg/kg every 4th day or vehicle detergent daily. Tumor volumes were measured every 2nd day and absolute volume was set relative to day 0. HCC827 xenografts showed reduction in tumor volumes of about 80% for the continuous_15mg/kg and the intermittent_4day schedules and tumor progression for the vehicle control ($p<0.001$) (left panel). PC9 xenografts showed a reduction of about 40% for the continuous_15mg/kg and the intermittent_4day schedules and tumor progression for the vehicle control ($p<0.001$) (middle panel). H1975 xenografts showed tumor progression for all schedules used (right panel).

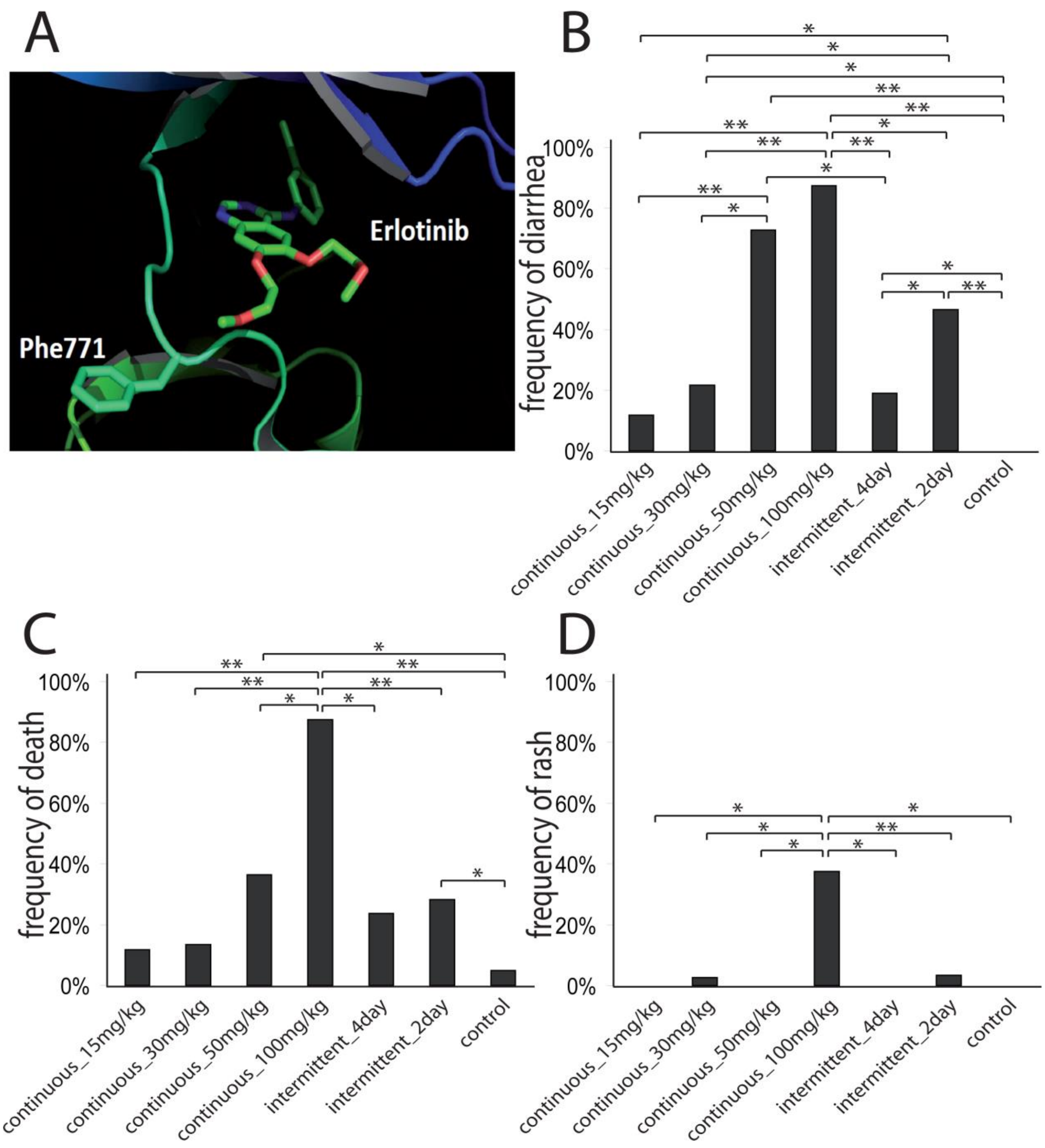

Figure S 4

A Erlotinib binding to the EGFR. Shown is the phenylalanine 771 beside the binding pocket which is replaced by a tyrosine in murine EGFR.

B – D Toxicity of erlotinib in mice: Shown is the frequency of toxic side effects in both tumor harboring and non-tumor harbouring mice treated with erlotinib 15mg/kg (n=18), 30mg/kg (n=37), 50mg/kg (n=11), 100mg/kg (n=8) daily or 200mg/kg every 4th (n=21) or every 2nd day (n=60) or with vehicle (n=20). *$p < 0.05$, **$p < 0.001$.

B Frequency of diarrhea

C Frequency of death

D Frequency of rash

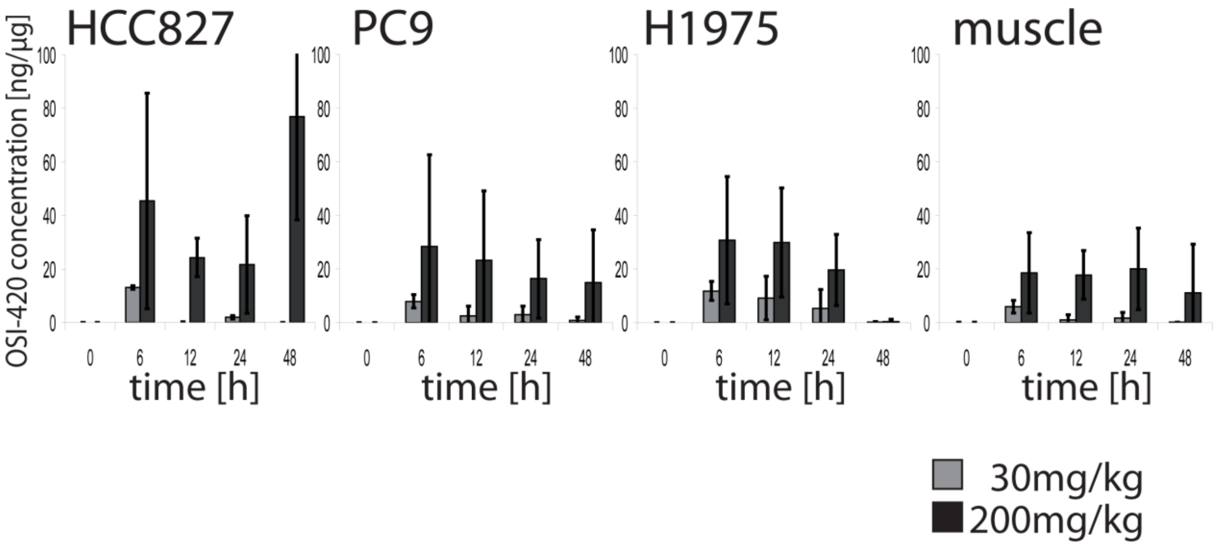

Figure S 5

Concentrations of OSI-420, an active metabolite of erlotinib, in tumor lysates or lysates of the right musculus quadriceps femoris, assessed by mass spectrometry in mice treated with a single dose of either 30mg/kg or 200mg/kg erlotinib. Lysates were prepared before therapy, 6, 12, 24 or 48 hours after administration of erlotinib. Erlotinib concentrations were set relative to the protein amount of the lysates. Mean data ±SD is shown.

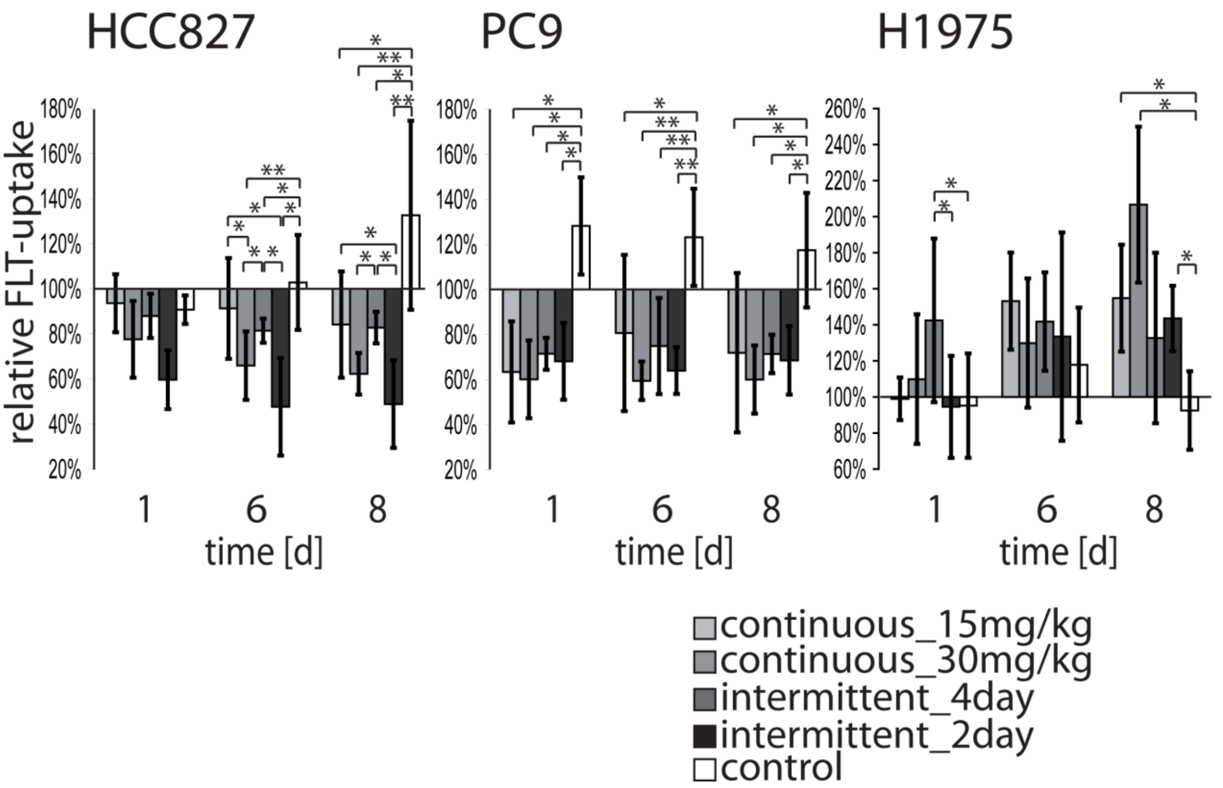

Figure S 6

Change in relative ^{18}F-FLT-uptake of xenografts (HCC827: left panel, PC9: middle panel, H1975: right panel). Mice were treated with erlotinib either 15mg/kg daily, 30mg/kg daily, 200mg/kg every 4th day, 200mg/kg every 2nd day or vehicle. ^{18}F-FLT-PET-imaging was performed the day before start of therapy (day -1), day 1, 6 and 8 after start of therapy. ^{18}F-FLT-uptake of tumors was set relative to the uptake of the mediastinum uptake and all values were set relative to day -1. *p < 0.05, **p < 0.001.

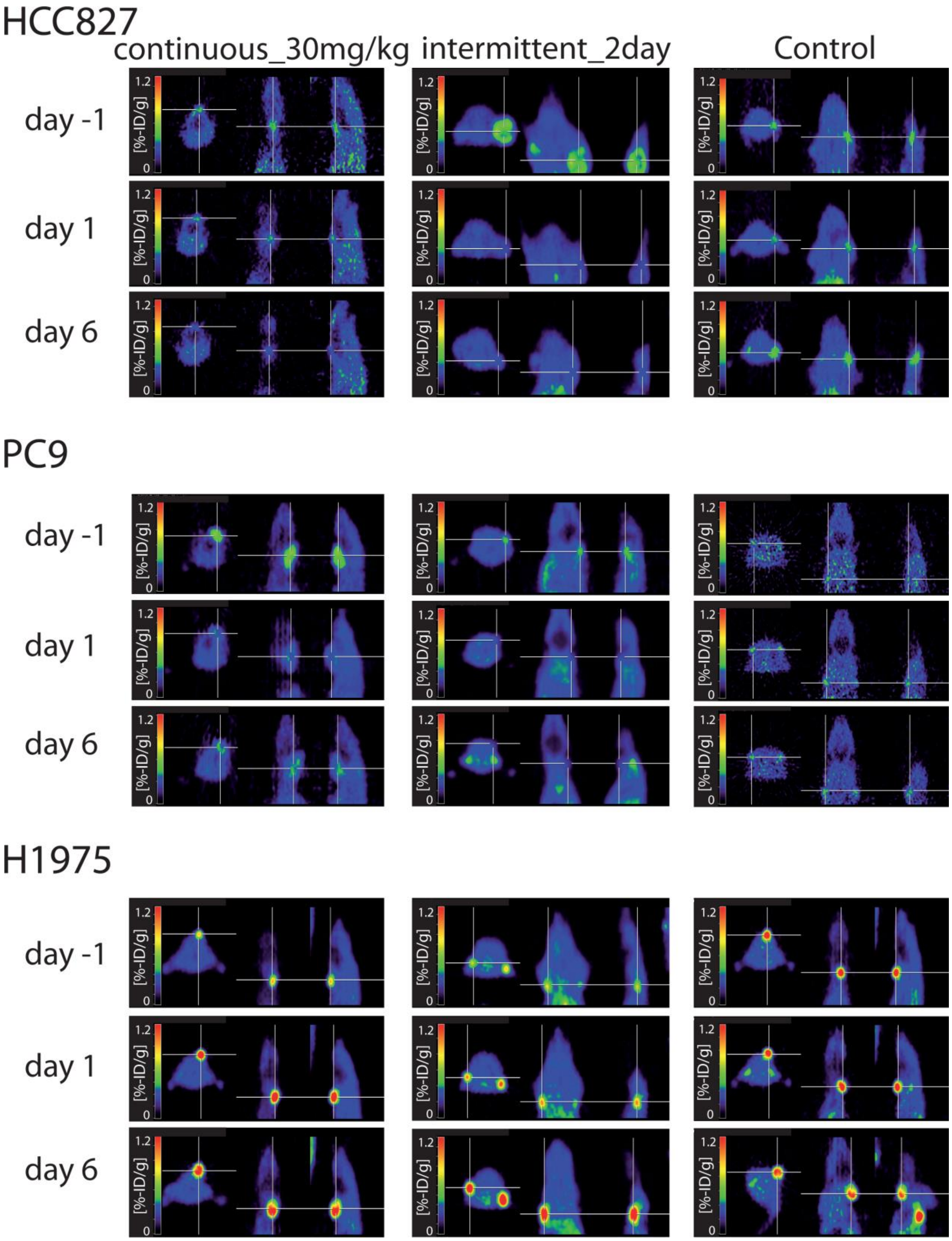

Figure S 7

Representative pictures of ^{18}F-FLT-PET of HCC827-, PC9- and H1975-xenografts treated with erlotinib 30mg/kg daily, 200mg/kg every 2nd day or vehicle, performed at day -1, 1 and 6. Cross hairs indicate tumor positions.

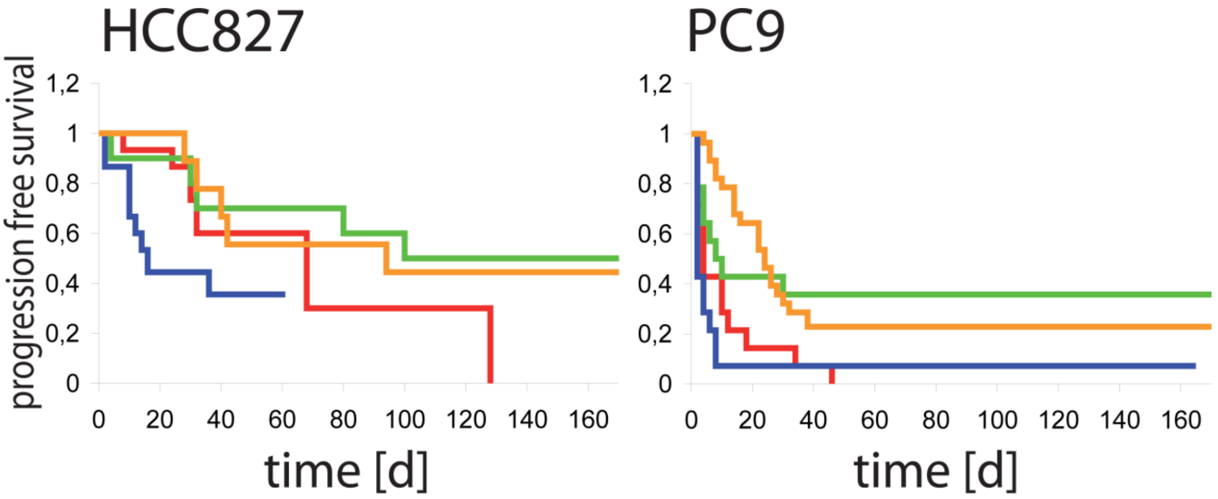

p-value	kont_30	HD_4d	HD_2d
kont_15	0.851	0.664	0.884
kont_30		0.661	0.996
HD_4d			0.786

p-value	kont_30	HD_4d	HD_2d
kont_15	0.592	0.846	0.0233
kont_30		0.308	0.981
HD_4d			0.000639

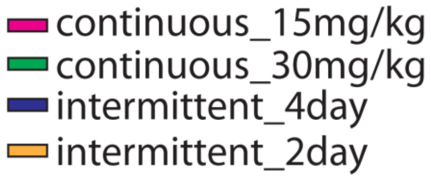

Figure S 8

Progression free survival (PFS) after stopping erlotinib therapy. Mice harboring HCC827- or PC9-xenografts were treated for 40 days with erlotinib 15mg/kg or 30mg/kg daily or 200mg/kg every 4th or every 2nd day. After therapy was stopped tumors were monitored for regrowth. Almost all tumors relapsed in the follow-up time. For HCC827 (left panel) the median PFS was 38 days for 15mg/kg daily (n=15), 125 days for 30mg/kg daily (n=10), 14 days for 200mg/kg every 4th day (n=15) and 94 days for 200mg/kg every 2nd day (n=9). However these differences were not significant. PC9-xenografts (right panel) had a median PFS of 4 days for 15mg/kg daily (n=14), 9 days for 30mg/kg daily (n=14), 2 days for 200mg/kg every 4th day (n=14) and 24 days for 200mg/kg every 2nd day (n=28). The PFS of the intermittent_2day was significantly longer than the continuous_15mg/kg schedule and the intermittent_4day schedule ($p<0.05$), however not superior to the continuous_30mg/kg schedule.

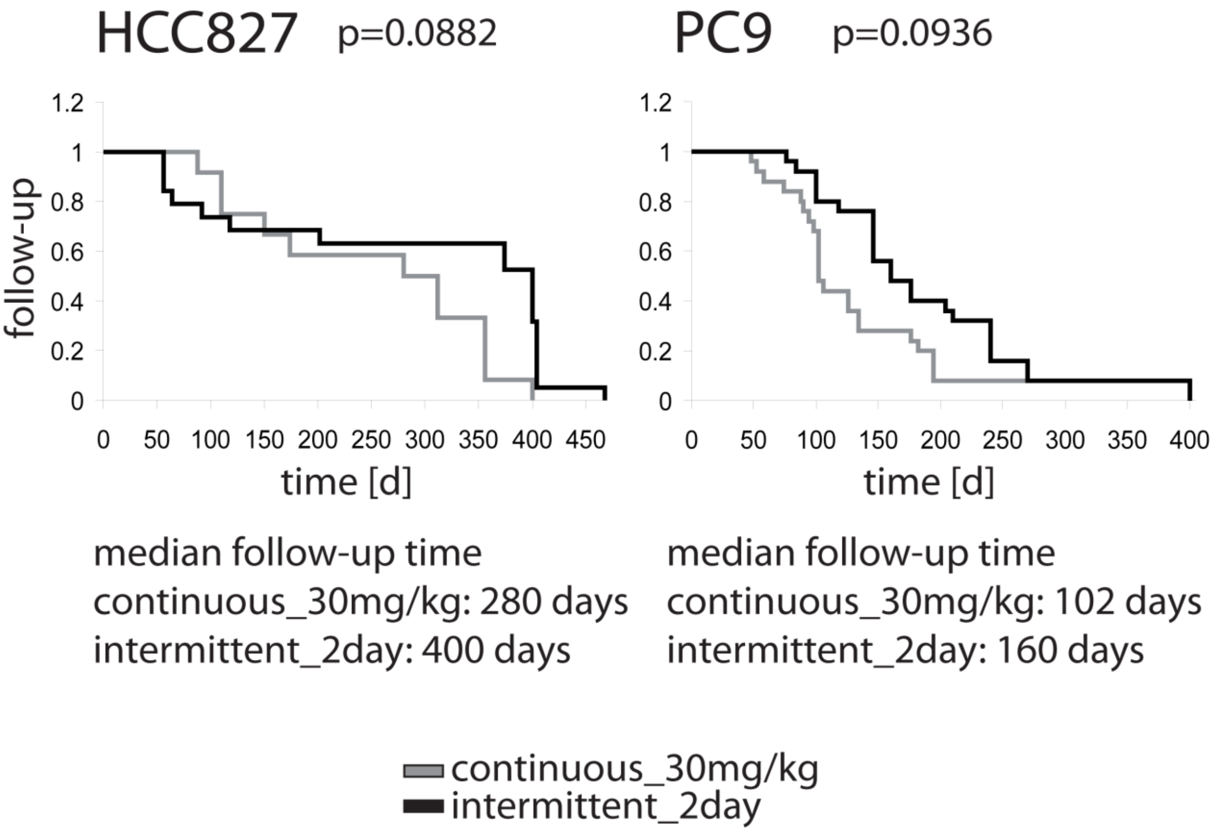

Figure S 9

Kaplan-Meyer curves of the follow-up time of long-term treated mice harboring HCC827-xenografts (left panel): n(continuous_30mg/kg) = 12, n(intermittent_2day) = 19, p=0.0882 and PC9-xenografts (right panel): n(continuous_30mg/kg) = 25, n(intermittent_2day) = 25, p=0.0936.

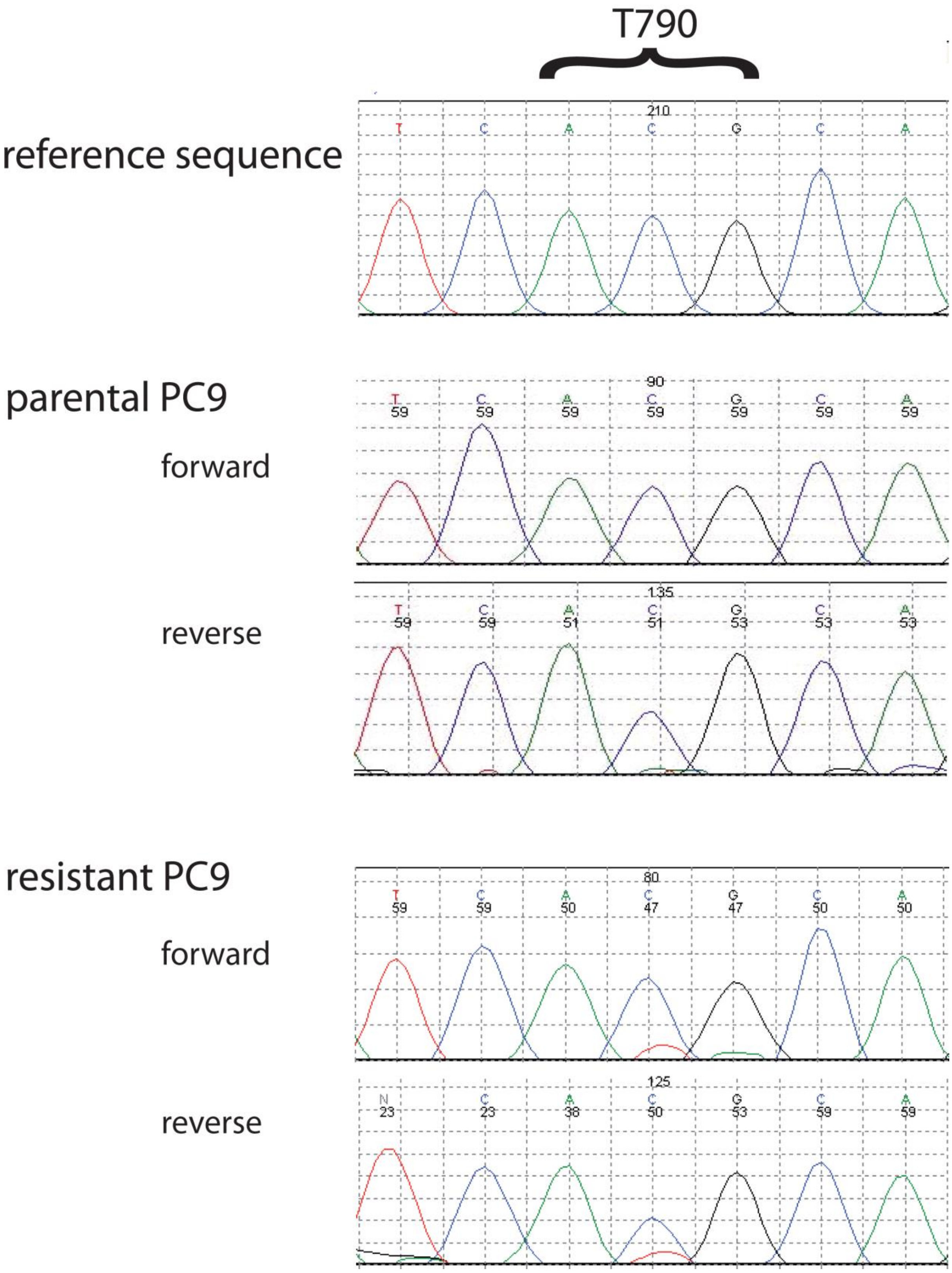

Figure S 10

T790M-mutation in PC9 cells with an in-vivo acquired erlotinib-resistance by sanger sequencing of EGFR exon 20. Shown are parental PC9-cells and PC9 with an acquired erlotinib resistance. The resistant PC9-tumors showed the T790M mutation by a C→T exchange.

Supplementary Methods

Cell-culture and reagents:

The human NSCLC-cell-lines PC9, HCC827 and H1975 were obtained from the American Type Culture Collection (ATCC), HCC827GR from the Jeff Engelman lab. Cells were cultured in RPMI-1640 medium with 10% FCS and 1% Penicillin+Streptomycin. All cell lines were grown as a monolayer at 37°C, in a humidified atmosphere of 5% CO_2 and 95% air.

Erlotinib was purchased from LC Labs, USA. For in-vitro studies erlotinib was prepared in stock solution of 10mM in DMSO (Sigma Aldrich, Germany) and stored at -20°C. For in-vivo studies erlotinib was dissolved in 6% Captisol® (CyDex Inc., USA) in concentrations of 10mg/ml (continuous schedules) or 30mg/ml (intermittent schedules) and stored in a rotating device at 4°C.

Western blotting:

For Western blot analyses 300,000 cells (HCC827, PC9, H1975 and HCC827GR) were seeded in 6 well plates and were incubated for 24 hours in RPMI-1640 medium. Then erlotinib (0.1µM, 1µM or 10µM) or DMSO was added and cells were treated for either 20 minutes or up to 48 hours. After 20 minutes cells were washed 3 times with PBS and cultured again in DMSO-containing RPMI or erlotinib respectively, till 48hours were complete. Cell lysates were prepared after 20 minutes, 3, 6, 12, 24 or 48 hours using cell lysis buffer (Cell Signaling) supplemented with protease (Roche) and phosphatase inhibitor (Calbiochem) cocktails. After 20 minutes of incubation on ice, lysates were centrifuged at 14,000 rpm for 30 minutes. Protein concentration in supernatants was measured using BCA Protein Assay (ThermoScientific). Equivalent amounts of protein (30µg) were denatured and separated on 4–12% SDS-PAGE gels and blotted on nitrocellulose membranes (Amersham Hybond-C Extra). Phosphorylation levels of the signal transduction cascade was determined using the following antibodies: pEGFR (Y1068), EGFR, pAkt (S473), AKT, pErk 44/42 (Thr202/Tyr204), ERK 44/42, Caspase-3 (Cell Signaling Technologies, USA) and ß-actin (clone C4) (MP Biomedicals LLC, USA). As secondary antibodies we used the anti-rabbit-HRP- and anti-mouse-HRP-antibody (Millipore, Germany). Each experiment was performed in triplicates.

Vitality Assay:

For MTT-Assays 3000 cells/well were plated in 96-well-plates and cultured for 24 hours in RPMI1640 medium (+10%FCS +1%P/S). Then erlotinib (0.1µM, 1µM or 10µM) or DMSO in 200µl medium was added and cells were treated for either 20 minutes or 72 hours. After 20

minutes cells were washed 3 times with PBS and cultured again in DMSO-containing RPMI or erlotinib respectively, till 72hours were complete.
Then 50µl MTT (5mg/ml) (Sigma Aldrich, Germany) was added, incubated for 1 hour at 37°C, then medium was removed and 50µl DMSO was added. After incubating for another 15 minutes at 37°C, analysis was performed with a Mithras LB 940 Plate Reader (Berthold Technologies, Germany). Analysis-time: 0.5s, lamp-energy: 14000, filter: 570nm. Each condition was repeated 6 times in 3 independent settings.

Annexin V-flow-cytometry:
For flow cytometry HCC827, PC9, H1975 and HCC827GR cells were plated in 6-well plates and incubated for 24 hours, and then treated with erlotinib (0.1µM, 1µM or 10µM) or DMSO for either 20 minutes or 24 hours. After 20 minutes cells were washed 3 times with cold PBS and cultured again in DMSO-containing RPMI or erlotinib respectively, till 24 hours were complete. Flow cytometry was performed using the AnnexinV-FITC Apoptosis Detection Kit I (BD Pharmingen, Germany) according to the manufactures protocol and measured by Gallios Flow Cytometer (Beckmann Coulter), detecting at least 100,000 events per probe. Data was evaluated by setting appropriate gates in Kaluza analysis software (Beckman Coulter) and induction of apoptosis was calculated as the difference between treated samples and DMSO control. Each experiment was performed in quadruplicates.

In-vivo experiments:
All animal procedures were approved by the local animal protection committee and the local authorities.
Xenograft-experiments were performed in 10 to 15 week old male athymic NMRI-nude-mice (Janvier, Europe) as described recently [1, 2].
Mice were hold in a 12 hour light-dark-cycle. They got water and standard food ad libitum.
For creation or xenografts, tumor cells were cultured as described before. For implantation they were resuspended in plain RPMI1640 in a concentration of 100*10^6 cells/ml.
5*10^6 cells were injected subcutaneously in mice, under 2.5% Isoflurane-anaesthesia.

Therapy was started when tumors reached a size of 100mm^3. They were treated by oral gavage of erlotinib in these schedules: 15mg/kg daily, 30mg/kg daily, 200mg/kg every 2nd day or 200mg/kg every 4th day or with 100µl vehicle-detergent alone daily.
At the end of the experiment mice were sacrificed by intra peritoneal injection of Ketamin/Xylazine (300/60 mg/kg) and tumors were resected and fixed in 4% formaldehyde for histology.

From the beginning of therapy tumor growth was monitored every 2nd day by calimetric measurement. Tumor volumes were calculated by the modified ellipsoid formula [V = 0.5 x (long diameter) x (short diameter)^2]. All absolute tumor volumes were set relative to day 0.

Tumors became resistant to erlotinib-therapy were resected under sterile conditions and recultured in RMPI1640-medium (+10%FCS, +1%P/S) or resected and fixed in 4% formaldehyde for subsequent analyses.

Definition of an *acquired resistance* was as follows: initial response under therapy and regrowth of more than +50% of the minimum volume, but at least +10mm^3.

Toxicity of erlotinib in mice

To make sure toxic side effects in mice are similar in mice than in humans, we aligned murine and human EGFR by ClustalW-alignment (http://www.ebi.ac.uk/Tools/msa/clustalw2) and modeled erlotinib binding to the ATP-binding pocket of the EGFR.

For finding the tolerable therapy schedules of erlotinib we treated a small set of non-tumor harboring mice with erlotinib and measured weight every 2nd day.

Additionally, toxic side effects (diarrhea, rash and death) in all treated mice were monitored.

Pharmacokinetics of erlotinib

For pharmacokinetic analyses we treated mice with a single dose of either 30mg/kg or 200mg/kg erlotinib. After 0.5, 1, 1.5, 2, 2.5, 3, 4, 5, 6, 7, 8, 9, 10, 11, 12, 14, 16, 18, 20, 22, 24, 30, 36, 42 and 48 hours we took blood samples from the tail-vein (50µl blood) added 5µl Heparin (5000IE/ml) (Ratiopharm, Germany), centrifuged the samples for 15 minutes at 4000 rpm at 4°C. Supernatant plasma was stored at -20°C till analysis.

Sample preparation for analysis After thawing, plasma samples (50 µl) were precipitated with 100 µl acetonitrile (ACN) for the analytical assay and the internal standard [1 µl sunitinib dissolved in ACN] was added. After thorough mixing for 10 s, samples were centrifuged for 10 min at room temperature at 14000 g. After centrifugation, the supernatant was transferred into screw-capped glass vials for liquid chromatography tandem mass spectrometry (LC-MS/MS) analysis. Quantification of erlotinib plasma concentrations were quantified on an API 5000 with QJet™ Ion Guide (Applied Biosystems Foster City, CA, USA). The effluent of LC system (500 µl/min; 1200 series; Agilent, Waldbronn, Germany) was delivered through a reversed-phase column (150 x 2 mm, 4 µm Synergy Hydro-RP 80A, Phenomenex, Torrance, CA, USA) eluted with solvent system consisting of water 60% CAN, 40% of water at pH=3 (pH value was achieved with acetic acid) to the mass spectrometer. Plasma concentrations of erlotinib and sunitinib were detected by positive electrospray ionization (ESI^+) in the positive multiple reaction monitoring mode (MRM). The most intense mass transition was used as quantifier; two

additional transitions were used as qualifier. The transitions being used were: 394.2 → 278.1, 394.2 → 336.2 and 394.2 → 75.0 for erlotinib and 399.3 →283.0, 399.3 →326.1 and 399.3 →138.1 for sunitinib. Erlotinib and sunitinib were eluted after 0.51 min and 0.52 min, respectively. The lower limit of quantification (LLOQ) for erlotinib was 18 ng/ml. Instrument control and data acquisition were performed with the Analyst 1.4.2 software (Applied Biosystems). Peak area ratios (analyte/internal standard) were used for quantification and calibration functions were calculated via a weighted (1/x) least squares linear regression. Erlotinib concentrations ranged from 18 to 2000 ng/ml. Within the validation procedure the intra- and interday precision and accuracy were evaluated by assaying three replicates of quality control samples at each quality control concentration on the same day and three consecutive days. Precision was measured by the intra- and interday RSD (relative standard deviation). Accuracy was assessed by comparison of the calculated mean concentrations with nominal concentrations of quality control samples. The assay accuracy was expressed as the RE (relative error), that is (calculated mean concentration – nominal concentration)/nominal concentration x 100%. Intraday precision was between 0.6% and 9.9%. Intraday accuracy was between −4.2% and 5.2 %. Interday precision was between 2.7% and 11.1%. Intraday accuracy was between −5.2% and 2.7 %. The matrix of the calibration samples was human plasma. Matrix effects of human plasma and mice plasma were excluded by comparing peak ratios of quality control samples prepared with human plasma to those prepared with mice plasma within the validation procedure. Full comparability was proven.

Estimation of pharmacokinetic parameters Plasma concentrations were evaluated using a population pharmacokinetic approach by non-linear mixed effect modelling with the software package NONMEM® 7.2.0 (ICON Development Solutions, Ellicott City, MD, USA) with a GNU Fortran 4.6 compiler (Free Software Foundation, Inc., Boston, MA, USA). The toolkit PLT Tools 4.6.8 (PLTsoft, San Francisco, CA, USA) served as an application programming interface to NONMEM to aid model development and evaluation. Graphical data visualization was performed using R VERSION 2.12.0 (R Foundation for Statistical Computing, Vienna, Austria). The samples of all mice were included in the model. The model was specified as a set of differential equations that were solved by using the NONMEM subroutine ADVAN6. AUC was calculated as DOSE/Clearance. Model development followed the standard approach, i.e. we started with the most simple model (first order absorption, first order elimination, no error terms, no covariates) and stepwise tried to expand the model if an significant improvement of the fit was achieved. The most suitable model to describe the data was a one compartment model with first order absorption and first order elimination, an additive error model was used. Inter individual variability was incorporated for clearance and absorption rate. More complex models and/ or the inclusion of potential covariates such as body weight did not improve the fit significantly.

Pharmacodynamics and Mass Spectrometry of erlotinib from tumor lysates

For pharmacodynamical analyses and determination of erlotinib concentration in the tumor, we implanted HCC827, PC9 and H1975 xenografts as described before. After tumors reached a size of about $100mm^3$ we treated them with a single dose of either 30mg/kg or 200mg/kg erlotinib. Mice were sacrifices 6, 12, 24 or 48 hours after treatment or without treatment. Then tumors were resected and either formaline fixed for IHC or lysed for determination the concentrations of erlotinib and OSI-420 within the tumor by mass spectrometry. To create lysates tumors were flash-frozen in liquid nitrogen, then they were lysed with a homogenizer in organ lysis buffer (400µl/tumor). Lysis puffer was composed like this:

-10 ml 0.5M Hepes pH 7.4

-11 ml 50% Methanol

-10 ml 1M NaF

-10 ml 100 mM Natrium-Orthovanadat

-4 ml 250 mM EDTA

-1 ml 5 M NaCl

-666 µl Aprotinin (Sigma)

-200 µl Benzamidin

-1ml PMSF in EtOH

Final volume was made up to 100 ml with distilled water. Before use, 1 tablet of protease inhibitor (Roche) was added per 10ml of lysis buffer. The lysates were centrifuged at 14000 rpm for 1 hour at 4ºC. Supernatant was collected and stored at -80°C till processing.

For absolute quantification of erlotinib and OSI-420 an Acquitiy UPLC (Waters) was connected to a Xevo™ TQ (Waters). An Acquity UPLC BEH C18 1.7µm, 2.1 x 50mm column was used at 25°C. Solvent A was 0.1% formic acid (Biosolve) and B acetonitrile (Biosolve). A linear gradient from 95% A to 5% in 4.10min at a flow rate of 0.4ml/min was used. 2µL of standard and 2µl of sample were injected. The sample manager was set to 6°C for the standards. The TQ was operated in positive ESI MRM (multi reaction monitoring) mode. The source temperature was set to 150°C, desolvation temperature was 650°C and desolvation gas was set to 800L/h, cone gas to 50L/h. The following MRM transitions were used for erlotinib m/z 394.03 $(M+H^+)^+$ to 277.95 (quantifier) collision energy 30V, m/z 394.03 to 303.95 (qualifier) collision 30V, m/z 394.03 to 335.94 (qualifier) collision 22V, cone was in all cases 39V, for OSI-420 m/z 380.03 to 277.85 (quantifier) collision energy 30V, m/z 380.03 to 249.89 (qualifier) collision 34V, m/z 380.03 to 321.93 (qualifier) collision 22, cone was in all cases 39V. As data management software MassLynx (Waters) and for data evaluation and absolute quantification TargetLynx

(Waters) were used. All compounds were fresh prepared during 2 months and dissolved in 0.1% Formic acid (Bisolove) prepared with 0.22μm MilliQ-Water. With erlotinib eluting at 2.94min a standard calibration curve was calculated using following concentrations: 0.2, 0.5, 1, 5, 20, 50, 150, 300, 500, 750 ng/ml (prepared individually from stock solutions 100μg/ml). With OSI-420 eluting at 2.51min a standard calibration curve was calculated using following concentrations: 0.1, 0.5, 1, 2, 4, 6, 8, 10 ng/ml (prepared individually from stock solutions 100μg/ml).
Correlation coefficient: $r < 0.990$; response type: external standard, area; curve type linear; weighting 1/x. The peak integrations were corrected manually, if necessary. Quality control standards of each standard were used during sample analysis and showed between 0.5% and 40% deviation respectively. Blanks after the standards, quality control and sample batch proved to be sufficient. No carry over was detected.
Erlotinib and OSI-420 concentration in the lysates were set relative to their protein concentration.

Immunohistochemistry:

Tumors were resected and fixed in 4% formaldehyde for 24 hours and transferred to PBS. Tissues were embedded in paraffin following standard protocol, 3 μm thick slices were cut and stained with primary antibodies as follows: Ki67 (1:50, pretreatment pH6 20min), pEGFR Tyr 1068 (1:100 over night, pretreatment pH6 20min), Cleaved Caspase3 (1:100, pretreatment pH6 20min). Corresponding secondary antibody detection kits for reduced background on murine tissue were used (Histofine Simple Stain Mouse MAX PO and Histofine mousestain kit, medac) and stained on an automated stainer (LabVision Autostainer 480S, Thermo Scientific).

^{18}F-FLT-PET:

Synthesis of [^{18}F]fluoro-L-thymidine (^{18}F-FLT) and PET measurement protocols were performed as described elsewhere [1, 3, 4].
Mice harboring HCC827, PC9 and H1975 xenografts were measured one day before start of therapy (day -1). Than at the second day of therapy (d=1), at day 6 and 8 using a R4 microPET scanner (Concord Microsystems, Inc, Knoxville, TN). Quantitative analysis was done using the in-house software VINCI and all data were decay corrected. We therefore used a region of interest (ROI) analysis of the axial cut of the tumor at its largest diameter, coregistering all subsequent measurements. Mean activity of each tumor was set relative to the mean activity of the mediastinum of the corresponding mouse. And all longitudinal measurements were set relative to day -1.

Sequencing for resistance mutations

To detect resistance mechanisms of resistant PC9 tumors, tumors were resected and cut into small pieces under sterile conditions. Tumors were lysed in trypsin and cells were incubated in RPMI-1640 medium with 10% FCS and 1% Penicillin+Streptomycin plus 1µM erlotinib. Cells were grown in cell culture flaskes for at least 3 passages. Then DNA of these cells was isolated using the Gentra Puregene Cell Kit following the manufacture protocol. By PCR using the Quiagen Hot start Polymerase EGFR exon 20 was enriched, followed by a cleanup-step using the ExoSAP IT-kit and samples were finally sequenced using the Big Dye polymerase and M13-forward- and -reverse-primers.

In total 5 resistant tumors were analyzed, 4 from the continuous_30mg/kg-group and 1 from HD_2d-group. T790M-mutations could be detected in all of them.

1. Ullrich, R.T., et al., *Early detection of erlotinib treatment response in NSCLC by 3'-deoxy-3'-[F]-fluoro-L-thymidine ([F]FLT) positron emission tomography (PET).* PLoS One, 2008. **3**(12): p. e3908.
2. Chatterjee, S., et al., *Tumor VEGF:VEGFR2 autocrine feed-forward loop triggers angiogenesis in lung cancer.* J Clin Invest, 2013. **123**(4): p. 1732-40.
3. Shields, A.F., et al., *Imaging proliferation in vivo with [F-18]FLT and positron emission tomography.* Nat Med, 1998. **4**(11): p. 1334-6.
4. Kobus, D., et al., *A fully automated two-step synthesis of an (18)F-labelled tyrosine kinase inhibitor for EGFR kinase activity imaging in tumors.* Appl Radiat Isot, 2009. **67**(11): p. 1977-84.
